# The Many Roles of Macrophages in Skeletal Muscle Injury and Repair

**DOI:** 10.3389/fcell.2022.952249

**Published:** 2022-07-11

**Authors:** Xingyu Wang, Lan Zhou

**Affiliations:** Department of Neurology, Boston University School of Medicine, Boston, MA, United States

**Keywords:** skeletal muscle, macrophage, injury repair, regeneration, satellite cells

## Abstract

Skeletal muscle is essential to physical activity and energy metabolism. Maintaining intact functions of skeletal muscle is crucial to health and wellbeing. Evolutionarily, skeletal muscle has developed a remarkable capacity to maintain homeostasis and to regenerate after injury, which indispensably relies on the resident muscle stem cells, satellite cells. Satellite cells are largely quiescent in the homeostatic steady state. They are activated in response to muscle injury. Activated satellite cells proliferate and differentiate into myoblasts. Myoblasts fuse to form myotubes which further grow and differentiate into mature myofibers. This process is tightly regulated by muscle microenvironment that consists of multiple cellular and molecular components, including macrophages. Present in both homeostatic and injured muscles, macrophages contain heterogeneous functional subtypes that play diverse roles in maintaining homeostasis and promoting injury repair. The spatial-temporal presence of different functional subtypes of macrophages and their interactions with myogenic cells are vital to the proper regeneration of skeletal muscle after injury. However, this well-coordinated process is often disrupted in a chronic muscle disease, such as muscular dystrophy, leading to asynchronous activation and differentiation of satellite cells and aberrant muscle regeneration. Understanding the precise cellular and molecular processes regulating interactions between macrophages and myogenic cells is critical to the development of therapeutic manipulation of macrophages to promote injury repair. Here, we review the current knowledge of the many roles played by macrophages in the regulation of myogenic cells in homeostatic, regenerating, and dystrophic skeletal muscles.

## 1 Introduction

Skeletal muscle injury can be acute or chronic depending on etiologies. Acute injury is commonly caused by trauma, ischemia, freeze, or myotoxin exposure. Chronic injury is usually associated with a disease process, such as muscular dystrophy, inflammatory myopathy, or infectious myopathy. Skeletal muscle injury repair is a complex process, consisting of muscle inflammation, regeneration, revascularization, and extracellular matrix (ECM) remodeling (Bentzinger et al., 2013a; Yin et al., 2013; Dumont et al., 2015a). As a tissue that constantly encounters mechanical stretch, skeletal muscle suffers a high rate of micro-injury in the normal steady state. To cope with this challenge, skeletal muscle has evolutionarily developed a remarkable regenerative capacity, which involves activation, proliferation, differentiation, and growth of myogenic cells (Bentzinger et al., 2013a; Yin et al., 2013; Dumont et al., 2015a). Acutely injured skeletal muscle repairs well if the injury is not large or repeated. The injury repair process, however, requires an adequate inflammatory response which is initiated by transient neutrophil infiltration followed shortly by massive macrophage infiltration. Infiltrating macrophages not only phagocytose damaged tissue debris but also produce cytokines and growth factors to interact with myogenic, fibrogenic, and angiogenic cells to support skeletal muscle injury repair (Tidball and Villalta, 2010; Tidball, 2011; Muñoz-Cánoves and Serrano, 2015; Dort et al., 2019). While essential to the acute skeletal muscle injury repair, infiltrating macrophages contribute to muscle pathology in chronic injury associated with muscular dystrophy. With such diverse roles, macrophages have become a central topic of research in the field of skeletal muscle injury repair.

Macrophages are heterogeneous and multi-functional cells that are critical to tissue functions in both steady state and disease state. Although classically identified as innate immune cells, functioning in the activation and resolution of tissue inflammation, it is now clear that macrophages play important roles in a much wider range of biological processes, such as tissue remodeling during organogenesis, tissue homeostasis, injury repair, and immune response to pathogens (Hashimoto et al., 2013; Wynn et al., 2013; Kierdorf et al., 2015; Ginhoux et al., 2016; Wynn and Vannella, 2016). In the normal steady state, resident macrophages maintain tissue homeostasis via surveillance of local tissue environment and response to physiological and pathological changes. In a disease state, macrophages exert pro-inflammatory, anti-inflammatory, pro-fibrotic, or pro-regenerative functions depending on the tissue environment and macrophage origin. They are critically involved in a variety of disease processes, such as chronic tissue inflammation, tumor growth and metastasis, and tissue fibrosis (McNelis and Olefsky, 2014; Noy and Pollard, 2014; Ginhoux et al., 2016; Wynn and Vannella, 2016; Vannella and Wynn, 2017). Such diverse capabilities of macrophages are rooted in their diverse origins and high plasticity when responding to environmental changes. In this article, we will review the multiple origins and many roles of macrophages in skeletal muscle homeostasis, regeneration following acute injury, and degeneration, regeneration, and fibrosis in muscular dystrophy.

## 2 Skeletal Muscle-Resident Macrophages Arise From Multiple Origins and Appear Active in Maintaining Tissue Homeostasis and Promoting Muscle Growth and Regeneration

Tissue macrophages consist of two classes: resident macrophages and infiltrating macrophages. In adult mammals, while resident macrophages are present in all tissues, infiltrating macrophages are found in a diseased tissue, such as injured tissue. Unlike infiltrating macrophages which are all derived from blood monocytes originating from bone marrow hematopoietic stem cells (HSCs), tissue-resident macrophages arise from multiple origins during embryonic and adult hematopoiesis.

Macrophages reside in homeostatic tissues including skeletal muscle (Wang et al., 2020). Most of the tissue-resident macrophage populations in the steady state are established prenatally by two embryonic progenitors: primitive yolk sac macrophages and fetal liver monocytes (aka fetal monocytes) (Ginhoux et al., 2010; Schulz et al., 2012; Hashimoto et al., 2013; Ginhoux and Jung, 2014; Gomez Perdiguero et al., 2015; Hoeffel et al., 2015; Hoeffel and Ginhoux, 2015; Mass et al., 2016; Hoeffel and Ginhoux, 2018). Primitive yolk sac macrophages originate from early erythro-myeloid progenitors (EMPs) which emerge in yolk sac at embryonic day 7 (E7) in mice. They differentiate into primitive macrophages and migrate to embryonic tissues from E9.5. Fetal monocytes mainly arise from late EMPs that emerge in yolk sac at E8.5. They migrate into fetal liver and differentiate into fetal monocytes at E12.5. Fetal monocytes seed all other embryonic tissues except for brain (Hoeffel et al., 2015). Within individual tissues, primitive macrophages and fetal monocytes are induced by local tissue environment to differentiate into tissue-specific resident macrophages, expressing tissue-specific transcription factors and displaying tissue-specific functions. They persist into adulthood through proliferative self-renewal. Pre-hematopoietic stem cells (HSCs) first appear at aorta-gonad-mesonephros (AGM) at E9.5 and then seed fetal liver around E10.5, where they differentiate into mature HSCs (Hoeffel and Ginhoux, 2015). HSCs also contribute to fetal monocytes at the later stage of embryonic development (Gomez Perdiguero et al., 2015; Wang et al., 2020). Mature HSCs migrate into nascent bone marrow at the late embryonic stage and give rise to blood monocytes (adult monocytes) after birth. Adult monocytes are recruited by many tissues to replenish resident macrophages (Tamoutounour et al., 2013; Bain et al., 2014; Epelman et al., 2014; Bain et al., 2016; Scott et al., 2016), including by skeletal muscle (Wang et al., 2020) but not the brain (Hoeffel et al., 2015). Depending on the origin and tissue environment, resident macrophages display high plasticity in their function and activation.

Resident macrophages in the steady-state skeletal muscle have been identified and studied in mice (Wang et al., 2020). CD45^+^F4/80^+^CD64^+^ resident macrophages are found in interstitial tissues of skeletal muscle, expressing a low level of Ly6C and a high level of CD163 and CD206. They arise from both embryonic hematopoietic progenitors, including yolk sac primitive macrophages and fetal liver monocytes, and adult bone marrow HSCs. The transcriptome of resident macrophages in skeletal muscle is highly distinctive from that in other tissues. Skeletal muscle-resident macrophages express a specific set of transcription factor genes, including *Maf*, *Mef2c*, and *Tcf4* (Wang et al., 2020). They appear active in maintaining tissue homeostasis and promoting muscle growth and regeneration based on their differentially expressed genes. Functionally diverse subsets correlating to their origins are identified within skeletal muscle-resident macrophages. While the CCR2^+^MHCII^hi^Lyve1^low^ macrophages are mainly derived from adult blood monocytes and are more active antigen-presenting cells, the CCR2^-^MHCII^low^Lyve1^hi^ macrophages arise from both embryonic and adult progenitors and are more active phagocytes. Both subsets may play roles in maintaining skeletal muscle homeostasis. Interestingly, skeletal muscle-resident macrophages also have muscle type-specific features, as they express a higher level of stress response genes in respiratory muscle than in limb muscle (Wang et al., 2020).

Functional study of skeletal muscle-resident macrophages is limited. A recent study showed that in multiple tissues, including skeletal muscle, tissue-resident macrophages can rapidly cloak tissue microlesions through sensing damage-associated alarmins. The cloaking prevents chemoattractant signaling-mediated neutrophil swarms and subsequent inflammatory tissue damage. As a result, tissue microlesions heal without inflammation (Uderhardt et al., 2019). In the steady-state skeletal muscle, the cloaking by resident macrophages prevents complete death of myofibers with microlesions and maintains their structural integrity (Uderhardt et al., 2019). The findings support the important homeostatic function of resident macrophages in the steady-state skeletal muscle. The interactions between resident macrophages and myogenic cells in the steady-state and the roles of resident macrophages in skeletal muscle development and postnatal growth remain largely unknown and need to be determined.

## 3 Infiltrating Macrophages are Essential to Skeletal Muscle Regeneration Following Acute Injury

### 3.1 Skeletal Muscle Regenerates Well Following Acute Injury

**FIGURE 1:**
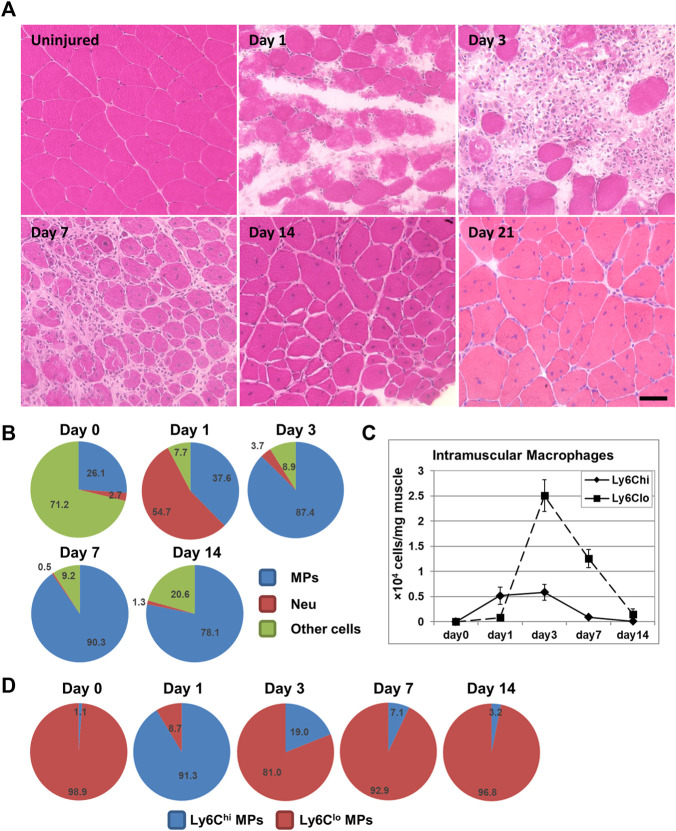
**(A)** H&E staining illustrating the normal acute muscle injury repair process following intramuscular BaCl_2_ injection into mouse quadriceps muscle. Bar = 50 μm. **(B)** Pie chart showing percentages of macrophages (MPs), neutrophils (Neu), and other cells among CD45^+^ cells in BaCl_2_-injured quadriceps muscle at different days post injury. **(C)** Line graph showing densities of intramuscular Ly6C^hi^ and Ly6C^lo^ macrophages in BaCl_2_-injured quadriceps at different days post injury. **(D)** Pie charts showing percentages of Ly6C^hi^ and Ly6C^lo^ subpopulations among macrophages in BaCl_2_-injured quadriceps at different days post injury.

Skeletal muscle has an excellent regenerative capacity. Unless caused by a repeated or a large volumetric muscle loss injury (Dadgar et al., 2014; Corona et al., 2015), acutely injured skeletal muscle regenerates well. Acute skeletal muscle injury can be caused by many etiologies, including intense exercise, trauma, ischemia, freeze, and myotoxin exposure. To study acute skeletal muscle injury repair, several animal models have been developed and used, including those with acute injuries caused by mechanical damage, intramuscular toxin or heavy metal salt injection, muscle freeze, and muscle ischemia induced by artery ligation. The technical merits of different injury models are reviewed by Baghdadi and Tajbakhsh (2018). Studies with these models have revealed a similar muscle repair process with small differences. Figure 1A illustrates the time course of murine skeletal muscle repair following acute injury induced by barium chloride. Massive muscle fiber necrosis is observed at day 1 post-injury, accompanied by inflammatory cell infiltration. The inflammation peaks at day 3. Small, central-nucleated myoblasts and multi-nucleated myotubes emerge around day 5. At day 7, infiltrating inflammatory cells drop significantly in number, and necrotic fibers are largely replaced by regenerating fibers. Extracellular matrix deposition is increased (transient fibrosis) at this stage to provide structural support to the injury repair. Inflammation and transient fibrosis resolve by day 14, and muscle fibers reach the size comparable to un-injured muscle.

### 3.2 Myogenic Cell Homeostasis, Activation, and Differentiation During Skeletal Muscle Regeneration

Regeneration of injured skeletal muscle relies on muscle-resident stem cells, satellite cells, as depletion of satellite cells completely abolishes skeletal muscle regeneration (Murphy et al., 2011). In the homeostatic healthy muscle, satellite cells are quiescent and in close association with myofibers, residing between sarcolemma of muscle fibers and basal lamina that surrounds fibers (Mauro, 1961). Quiescent satellite cells are characterized by the expression of paired box 7 (Pax7) (Seale et al., 2000) and forkhead box (FOXO) transcription factors (García-Prat et al., 2020). Upon injury, satellite cells undergo activation and differentiation to regenerate muscle. This process is regulated by a distinct set of transcription factors termed myogenic regulatory factors (MRFs) which include MYOD, MYF5, MRF4 (MYF6), and myogenin (MYOG) (Segalés et al., 2015; Hernández-Hernández et al., 2017; Massenet et al., 2021). *Myf5* and *Myod1* are transcribed in quiescent satellite cells, but the subsequent protein translation is prevented by posttranscriptional regulation (Beauchamp et al., 2000; Crist et al., 2012; van Velthoven et al., 2017; Yue et al., 2020). Upon muscle injury, the injured muscle microenvironment releases signals to activate satellite cells to allow protein translation of *Myf5* and *Myod1* mRNAs (Crist et al., 2012; Yue et al., 2020) and loss of FOXO expression (García-Prat et al., 2020). Activated satellite cells can generate both Pax7^+^MYF5^+^ and Pax7^+^MYF5^−^ cells through apical-basal asymmetric division, of which the Pax7^+^MYF5^+^ cells exhibit precocious differentiation, while the Pax7^+^Myf5^−^ cells contribute to the satellite cell reservoir (Kuang et al., 2007). Activated Pax7^+^MYF5^+^MYOD^+^ satellite cells, which are also called myoblasts, expand through symmetric division. Terminal differentiation of myoblasts, symbolled by upregulation of MYOG and MYF6/MRF4 and loss of PAX7 and MYF5 expression, generates myocytes and ultimately myofibers through fusion (Bentzinger et al., 2013a; Yin et al., 2013; Dumont et al., 2015a; Hernández-Hernández et al., 2017; Massenet et al., 2021).

The dynamic balance among quiescence, activation, and differentiation of satellite cells is vital to the maintenance of stem cell pool in healthy muscle and the successful regeneration in injured muscle. It is tightly regulated during skeletal muscle regeneration following injury (Dumont et al., 2015b). Infiltrating macrophages contribute to the signals required for satellite cell activation and differentiation.

### 3.3 Macrophages are the Predominant Inflammatory Cells in Acutely Injured Muscle, and They Differentiate From Circulation-Derived Inflammatory Monocytes

Although multiple immune cells are involved in the inflammatory response induced by acute muscle injury, neutrophils and macrophages are the predominant ones (Figure 1B) (Tidball and Villalta, 2010; Tidball, 2011; Yin et al., 2013). Neutrophils are the earliest inflammatory cells that infiltrate injured muscle. The neutrophil infiltration starts within 2 h post-injury, and the number peaks around 24 h post-injury (Tidball and Villalta, 2010). Neutrophils phagocytose damaged muscle debris and release reactive oxygen species (ROS), protease, and inflammatory cytokines to promote inflammation (Tidball, 2011; Wang et al., 2018). Depleting neutrophils during acute skeletal muscle injury impairs phagocytosis of necrotic tissue and delays regeneration (Teixeira et al., 2003; Toumi et al., 2006). Ly6C^hi^ monocyte/macrophage infiltration starts shortly after the neutrophil infiltration, and the number peaks 1–3 days after injury (Figures 1B–D) (Arnold et al., 2007). The total number of macrophages and the number of Ly6C^lo^ macrophages peak at day 3 post-injury (Figures 1B–D) (Arnold et al., 2007; Wang et al., 2018). Inflammation resolution is complete by day 14 (Arnold et al., 2007; Wang et al., 2018).

Infiltrating macrophages are derived from blood inflammatory monocytes (Arnold et al., 2007; Shi and Pamer, 2011). Blood monocytes consist of two principal subsets: Ly6C^hi^CCR2^+^CX3CR1^lo^ and Ly6C^lo^CCR2^−^CX3CR1^hi^ cells in mice, distinguished by the expression of cell surface markers Ly6C, C-C motif chemokine receptor 2 (CCR2), and CX3C chemokine receptor 1 (CX3CR1) (Geissmann et al., 2003). Tissue recruitment of cells from blood circulation requires the chemokine system, with tissue cells expressing chemokine ligands to chemoattract blood cells that express corresponding chemokine receptors. The Ly6C^hi^CCR2^+^CX3CR1^lo^ cells are inflammatory monocytes, which rapidly enter tissues upon injury or infection and differentiate into inflammatory macrophages (Geissmann et al., 2003). It has been shown that spleen is also a reservoir of the Ly6C^hi^ inflammatory monocytes which can be deployed into inflamed tissues, including skeletal muscle (Swirski et al., 2009; Rizzo et al., 2020). The recruitment of Ly6C^hi^ inflammatory monocytes by acutely injured muscle requires CCR2 expression by monocytes and CC chemokine ligand 2 (CCL2) expression by both muscle resident cells and infiltrating macrophages (Sun et al., 2009; Lu et al., 2011a; Lu et al., 2011b). CCL2 is the main ligand of CCR2. Deficiency in CCR2 or CCL2 diminishes macrophage infiltration in several acute skeletal muscle injury models (Arnold et al., 2007; Contreras-Shannon et al., 2007; Sun et al., 2009; Lu et al., 2011a; Lu et al., 2011b). The Ly6C^lo^CCR2^−^CX3CR1^hi^ cells are patrolling monocytes, which patrol the vascular endothelial surface and may enter tissue *via* CX3CR1/CX3CL1 to contribute to tissue-resident macrophages (Auffray et al., 2007). It has been shown that Ly6C^lo^ monocytes are not recruited by acutely injured muscle during injury repair (Varga et al., 2013). However, while most of the intramuscular macrophages at day 1 are Ly6C^hi^, the majority at day 3 are Ly6C^lo^ (Figures 1C,D). The accumulation of Ly6C^lo^ macrophages is resulted from Ly6C^hi^-to-Ly6C^lo^ switch, as Ly6C^hi^ inflammatory macrophages switch into Ly6C^lo^ macrophages after phagocytosing necrotic muscle debris (Arnold et al., 2007). Ly6C is not expressed in human cells. The CD14^hi^CD16^low^ and CD14^low^CD16^hi^ monocytes in humans correspond to the Ly6C^hi^ and Ly6C^lo^ monocytes in mice, respectively (Ziegler-Heitbrock et al., 2010).

### 3.4 Macrophages Undergo Phenotype Changes With Time to Support Acute Skeletal Muscle Injury Repair

Macrophages have been historically classified into M1 (classically activated) and M2 (alternatively activated) subsets, mainly based on *in vitro* studies and *in vivo* studies of parasite infections (Martinez and Gordon, 2014). M1 and M2 macrophages are different in their activation stimuli, cell surface markers, arginine metabolism, and cytokine production profiles (Martinez et al., 2006; Martinez and Gordon, 2014). While M1 macrophages, activated by IFN-γ ± LPS, are pro-inflammatory, M2 macrophages, activated by Il-4 ± IL-13, can be anti-inflammatory, pro-regenerative, and/or pro-fibrotic. Following this bipolar macrophage activation model, the Ly6C^hi^ and Ly6C^lo^ macrophages in injured skeletal muscle have once been considered M1 and M2 macrophages, respectively, based on the findings that the Ly6C^hi^ macrophages express more pro-inflammatory genes, while the Ly6C^lo^ macrophages express more anti-inflammatory genes (Arnold et al., 2007; Ruffell et al., 2009; Perdiguero et al., 2011; Wang et al., 2014). However, there has been growing evidence demonstrating that the M1/M2 paradigm of macrophage activation is over-simplistic and cannot mimic complex *in vivo* settings, in which the macrophage activation status can be influenced by many other co-existing cell types. *In vivo*, M1 and M2 stimuli often co-exist, macrophages can display mixed M1/M2 phenotypes, and they may not expand clonally to maintain phenotype (Martinez and Gordon, 2014; Murray et al., 2014; Ransohoff, 2016). The phenotype of *in vivo* macrophages may be M1-like or M2-like but not strictly M1 or M2. Gene expression profiles of macrophages in acutely injured skeletal muscle indicate that the Ly6C^hi^ macrophages at an early stage of inflammation (day 1–2 post-injury) are not strictly M1, and the Ly6C^lo^ macrophages at the late stage of inflammation are not strictly M2 (Novak et al., 2014; Varga et al., 2016a; Wang et al., 2018). One study showed that although the Ly6C^hi^-to-Ly6C^lo^ switch of macrophages was accompanied by downregulation of M1 genes (*tnfa, il1b,* and *il6*) and upregulation of M2 genes (*cd206, tgfb1*, and *igf1*), the Ly6C^hi^ macrophages at day 1 co-expressed a high level of both M1 (*tnfa*, *il1b*, and *il6*) and M2 genes (*arginase 1*, *ym1*, and *il10*), and the Ly6C^hi^ and Ly6C^lo^ macrophages at day 3 expressed a similar level of many M1 and M2 genes (Wang et al., 2018). A more profound transcriptome analysis revealed four macrophage activation statuses, specifying the sequential changes of macrophages during acute muscle injury repair: 1) infiltrating Ly6C^hi^ macrophages expressing acute-phase proteins and exhibiting an inflammatory profile; 2) metabolic changes in macrophages characterized by decreased glycolysis and increased tricarboxylic acid cycle/oxidative pathways; 3) Ly6C^lo^ macrophages actively proliferating; 4) restorative Ly6C^lo^ macrophages featuring secretion of molecules for intercellular communication (Varga et al., 2016a). Both studies suggest that the changes in macrophage phenotype in injured muscle is driven by the changes in muscle microenvironment with time (Varga et al., 2016a; Wang et al., 2018). The macrophages at the early stage of inflammation display a more “pro-inflammatory” phenotype, while the macrophages at the later stage of inflammation display a more “anti-inflammatory” and “pro-regenerative” phenotype. This is further supported by the changes in macrophage-produced lipid mediators from pro-inflammatory lipids at the early-stage to pro-resolving lipids at the late-stage (Scher and Pillinger, 2005; Giannakis et al., 2019).

### 3.5 Macrophages Play Essential Roles in Supporting Acute Skeletal Muscle Injury Repair

An adequate inflammatory response predominated by macrophage infiltration is essential to acute skeletal muscle injury repair. The absence of macrophage infiltration or disruption of macrophage functions leads to profound impairment of muscle regeneration and development of muscle fibrosis (Arnold et al., 2007; Contreras-Shannon et al., 2007; Sun et al., 2009; Lu et al., 2011a; Lu et al., 2011b; Wang et al., 2014; Dort et al., 2019). Macrophages regulate not only inflammation but also the other aspects of injury repair, including muscle regeneration, ECM remodeling, and angiogenesis.

#### 3.5.1 Inflammation

Macrophages are both effectors and regulators of the inflammatory response after acute skeletal muscle injury. Ly6C^hi^ monocytes/macrophages massively infiltrate into injured muscle shortly after an injury occurs, and they produce a relatively high level of pro-inflammatory cytokines such as TNF-α (Arnold et al., 2007; Shi and Pamer, 2011; Wang et al., 2018). Pro-inflammatory cytokines can promote inflammation by increasing tissue damage and amplifying inflammatory cell recruitment (Arnold et al., 2007; Shi and Pamer, 2011). Pro-inflammatory macrophages are also known as active phagocytes, as they phagocytose and clear damaged tissue debris for muscle injury repair (Tidball and Villalta, 2010; Tidball, 2011). When macrophage infiltration is diminished due to CCR2 deficiency, the clearance of necrotic muscle fibers is protracted (Lu et al., 2011b). The necrotic fibers eventually disappear more likely by necrotic fiber autolysis than by macrophage phagocytosis in this setting. Phagocytosis of dead cells has been shown essential to the pro-to anti-inflammatory phenotype switch in macrophages (Arnold et al., 2007; Johann et al., 2007; Xiao et al., 2008; Mounier et al., 2013; Zhang et al., 2019; Saclier et al., 2020). The anti-inflammatory macrophages contribute, in part, to inflammation resolution. They express a variety of anti-inflammatory cytokines, such as IL-4, TGF-β1, and IGF-1 (Serhan and Savill, 2005; Arnold et al., 2007; Ruffell et al., 2009; Perdiguero et al., 2011; Mounier et al., 2013; Wang et al., 2014; Wynn and Vannella, 2016; Vannella and Wynn, 2017; Wang et al., 2018), as well as pro-resolving lipids (Scher and Pillinger, 2005; Giannakis et al., 2019). These anti-inflammatory molecules counteract pro-inflammatory signals, reduce ROS production, block neutrophil recruitment, and promote neutrophil apoptosis and clearance by macrophages (Serhan and Savill, 2005). Prolonged presence of neutrophils was observed in injured muscle when macrophages were depleted (Dumont and Frenette, 2010). Therefore, macrophages play essential roles in both initiation and resolution of inflammation during acute skeletal muscle injury repair.

#### 3.5.2 Muscle Regeneration

Macrophages interact with myogenic cells to regulated muscle regeneration following acute injury. In a study using *in vitro* engineered model of rat adult skeletal muscle repair, incorporation of macrophages was required to stimulate satellite cell-mediated myogenesis (Juhas et al., 2018). Implantation of macrophages within engineered tissues in a mouse dorsal window-chamber model augmented muscle regeneration and contractile function (Juhas et al., 2018). Meanwhile, macrophages of different activation status have been shown to differentially regulate myogenic cell activation, proliferation, and differentiation. While pro-inflammatory macrophages promote myoblast proliferation but inhibit myoblast fusion and differentiation, anti-inflammatory macrophages inhibit myoblast proliferation but promote myotube formation and differentiation (Arnold et al., 2007; Bencze et al., 2012; Saclier et al., 2013; Hsieh et al., 2018). The differential regulation is mediated, at least in part, by paracrine cytokines and growth factors released by macrophages. Fibronectin, an ECM component that is highly expressed by day 1 pro-inflammatory macrophages (Wang et al., 2018), can activate satellite cells (Bentzinger et al., 2013b). Pro-inflammatory macrophages also produce a high level of IL-6 (Zhang et al., 2013), TNF-α (Li, 2002), PGE2 (Ho et al., 2017), and A Disintegrin-Like and Metalloproteinase with Thrombospondin Type 1 Motif (ADAMTS1) (Du et al., 2017) that can stimulate satellite cell proliferation. On the other hand, molecules that are highly expressed by day 3 anti-inflammatory macrophages, including IL-4 (Horsley et al., 2003), IGF-1 (Dumont and Frenette, 2010; Lu et al., 2011b; Tonkin et al., 2015), and GDF-3 (Varga et al., 2016b), can stimulate myoblast differentiation and myofiber growth. The increase in glutamine synthesis in macrophages during pro-to anti-inflammatory phenotype transition can also boost satellite cell activation and muscle regeneration (Shang et al., 2020). Therefore, the pro-to anti-inflammatory macrophage phenotype switch is likely important for the sequential activation, proliferation, differentiation, and growth of myogenic elements to complete muscle regeneration for injury repair. The critical role of the spatiotemporal presence of pro- and anti-inflammatory macrophages in acutely injured muscle has been corroborated by *in vivo* studies showing that targeting signaling molecules that regulate the pro-to anti-inflammatory macrophage phenotype switch, including IGF-1 (Tonkin et al., 2015), Meteorin-like (Metrnl) (Baht et al., 2020), AMP-activated protein kinase-1 (AMPKα1) (Mounier et al., 2013; McArthur et al., 2020), Nuclear Factor IX (Nfix) (Saclier et al., 2020), CCAAT/enhancer binding protein-β (C/EBPβ) (Ruffell et al., 2009), and peroxisome proliferator-activated receptor-γ (PPARγ) (Varga et al., 2016b), impaired myofiber growth without affecting clearance of necrotic tissue. Direct physical contact of macrophages with myogenic cells also appears important for myogenesis, as *in vitro* co-culture experiments showed that physical contact of macrophages with myogenic cells prevented apoptosis of myogenic cells (Chazaud et al., 2003; Sonnet et al., 2006). Both paracrine and direct physical contact require close proximity between macrophages and myogenic cells, which has been observed *in vivo* (Saclier et al., 2013; Ceafalan et al., 2018). In regenerating muscle, pro-inflammatory macrophages are in close proximity to proliferating satellite cells, while anti-inflammatory macrophages are close to the area containing differentiated myoblasts (Saclier et al., 2013).

#### 3.5.3 Extracellular Matrix remodeling

A well-regulated ECM remodeling is important to providing structural support for skeletal muscle injury repair. The ECM components, collagen 6a (Col6a) (Urciuolo et al., 2013) and fibronectin (Bentzinger et al., 2013b), were also important to satellite cell activation. Fibro/adipogenic progenitors (FAPs), the effector cells of ECM remodeling, not only produce ECM proteins but also support satellite cell activation and differentiation to facilitate muscle regeneration (Joe et al., 2010; Uezumi et al., 2010; Murphy et al., 2011; Uezumi et al., 2014). When the regenerative process is impaired; however, FAPs drive fibro-fatty replacement and fail to support satellite cell activation (Uezumi et al., 2014). Therefore, the FAP activity and ECM remodeling must be properly regulated. Macrophages regulate the accumulation and activation of FAPs during acute skeletal muscle injury repair. Pro-inflammatory macrophages limit the accumulation of FAPs by secreting TNF-α to induce FAP apoptosis (Lemos et al., 2015). Anti-inflammatory macrophages, on the other hand, can promote activation of fibrogenic cells by producing a high level of pro-fibrotic factors, including TGF-β1, PDGFα, and PDGFβ (Wang et al., 2016). Anti-inflammatory macrophages, therefore, may contribute to the transient fibrosis during muscle injury repair. The importance of macrophage regulation of FAP activity and ECM remodeling is supported by the findings that depleting macrophages or blocking macrophage recruitment leads to muscle fibrosis (Lu et al., 2011a; Lemos et al., 2015).

#### 3.5.4 Angiogenesis

Revascularization to restore blood supply is vital for tissue injury repair. The exact regulatory roles played by macrophage functional subtypes in this process remain elusive (Rahat et al., 2014). There are mixed reports of the roles of infiltrating macrophages in angiogenesis or vascular remodeling during acute skeletal muscle injury repair, which could be due to the different injury models used in these studies. One study showed that the diminished macrophage infiltration caused by CCR2 deficiency delayed VEGF production and angiogenesis during the repair of cardiotoxin-injured muscle (Ochoa et al., 2007). But another study using the BaCl_2_ injury model showed that blocking macrophage recruitment did not affect endomysial capillary density (Lu et al., 2011b). It has also been shown in the cardiotoxin injury model that macrophage depletion caused a significant endothelial-to-mesenchymal transition of the endothelial-derived progenitors, compromised blood vessel formation, and increased collagen deposition (Zordan et al., 2014). In addition, restorative macrophages have been shown to stimulate interaction between angiogenic cells and myogenic cells *via* oncostain M production to couple angiogenesis and myogenesis during muscle regeneration (Latroche et al., 2017). The roles of macrophages in angiogenesis during acute skeletal muscle injury repair need to be further elucidated.

In summary, adequate macrophage infiltration is essential to acute skeletal muscle injury repair. Infiltrating macrophages actively interact with myogenic cells to regulate their activation, proliferation, differentiation, and growth for proper muscle regeneration. The sequential presence of pro- and anti-inflammatory macrophages is crucial to the tightly-regulated, satellite cell-mediated regenerative process. Insufficient macrophage infiltration or disrupted pro-to anti-inflammatory macrophage transition impairs muscle regeneration.

## 4 Macrophages Play Pleiotropic Roles in Chronically Injured Skeletal Muscles in Muscular Dystrophy

Chronically injured skeletal muscle features chronic inflammation, with continuous Ly6C^hi^ monocyte and macrophage infiltration and Ly6C^hi^-to-Ly6C^lo^ switch. This creates an asynchronous regenerative environment that interrupts the spatiotemporal presence of pro- and anti-inflammatory macrophages, leading to dysregulated muscle regeneration. This hypothesis is supported by a study utilizing a simplified model of repeated muscle injury (Dadgar et al., 2014). In this study, skeletal muscle injury was induced twice, separated by 4 or 10 days. Concurrent accumulation of both pro- and anti-inflammation macrophages was observed in injured muscle, along with the development of persistent inflammation and fibrosis and the impairment of muscle regeneration. The asynchronous microenvironment in chronically injured muscle is much more complex than this simplified model, which may drive macrophages to play very different roles. The most studied muscle disease caused by chronic injury is Duchene Muscular Dystrophy (DMD). DMD is a genetic disease caused by a defective dystrophin gene on the X chromosome, which leads to muscle membrane instability, muscle necrosis, secondary muscle inflammation and fibrosis, muscle weakness, and premature death (Hoffman et al., 1987; Emery, 1993). The most commonly used animal model for studying DMD is *mdx* mice.

### 4.1 Chronic Inflammation in Mdx Mice Is Predominated by Macrophage Infiltration

**FIGURE 2:**
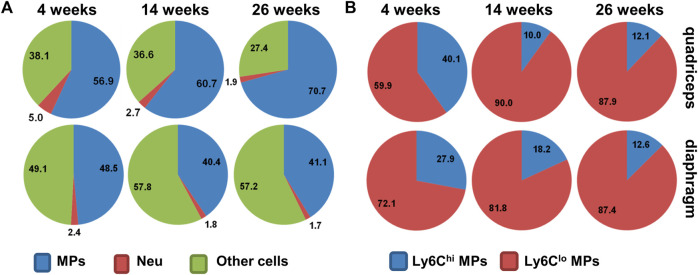
**(A)** Pie chart showing percentages of macrophages (MPs), neutrophils (Neu), and other cells among intramuscular CD45^+^ cells in *mdx* ^*5cv*^ mice at different ages. **(B)** Pie chart showing percentages of Ly6C^hi^ and Ly6C^lo^ subpopulations among intramuscular macrophages in *mdx* ^*5cv*^ mice at different ages.

*Mdx* or *mdx* ^*5cv*^ mice display a mild phenotype compared to DMD patients. But they do show persistent inflammation and progressive fibrosis in the diaphragm (Stedman et al., 1991; Goldspink et al., 1994; Hartel et al., 2001; Beastrom et al., 2011). Muscle inflammation in the *mdx* mice starts around age 3 weeks, persists into 2–3 months, and then subsides spontaneously in the limb muscles but the not the diaphragm. Progressive fibrosis mainly occurs in the diaphragm, which impairs respiratory function, resembling dystrophic muscles in human DMD patients (Stedman et al., 1991; Dupont-Versteegden and McCarter, 1992; Zhou et al., 2006; Huang et al., 2011). Muscle inflammation in *mdx* mice is also predominated by macrophage infiltration (Zhou et al., 2006) (Figure 2).

### 4.2 Macrophages Play Pleiotropic Roles in Dystrophic Muscles of Mdx Mice

Like in acute injury, muscle recruitment of Ly6C^hi^ inflammatory monocytes/macrophages in *mdx* is also mediated by CCR2, and intramuscular Ly6C^hi^-to-Ly6C^lo^ macrophage switch also occurs (Mojumdar et al., 2014; Zhao et al., 2017). Correspondingly, macrophages in the *mdx* leg muscles are more pro-inflammatory at 4 weeks while more pro-regenerative at 12 weeks (Villalta et al., 2009). Macrophages in *mdx* muscles appear pathogenic in general, as blocking Ly6C^hi^ inflammatory monocyte/macrophage infiltration *via* removal of splenic source of Ly6C^hi^ monocytes by splenectomy or *via* genetic ablation or pharmacological inhibition of CCR2 reduced muscle damage and fibrosis and improved muscle function in both leg and diaphragm muscles before 3 months of age (Mojumdar et al., 2014; Zhao et al., 2017; Rizzo et al., 2020). Similarly, toll-like receptor 4 (TRL4) deficiency in *mdx* mice, which also reduced macrophage infiltration at 6 and 12 weeks of age, decreased muscle fibrosis (Giordano et al., 2015). Macrophages may influence muscle inflammation, necrosis, regeneration, and fibrosis by producing pro-inflammatory, anti-inflammatory, pro-regenerative, and pro-fibrotic cytokines and growth factors, such as iNOS, TNF-α, IL-1β, IL-6, IL-10, IGF-1, TGF-β, and osteopontin (Zhou et al., 2006; Villalta et al., 2009; Tidball and Villalta, 2010; Villalta et al., 2011; Lemos et al., 2015; Capote et al., 2016; Ji et al., 2020).

In *mdx* limb muscles, the pro-inflammatory macrophages appear to contribute to muscle damage, as depleting macrophages by an F4/80 neutralizing antibody reduced leg muscle necrosis at 4 weeks of age (Wehling et al., 2001). The anti-inflammatory macrophages appear more pro-regenerative than pro-degenerative, which may contribute to the remarkable spontaneous improvement of limb muscle pathology after 3 months of age (Zhou et al., 2006; Beastrom et al., 2011). This hypothesis is supported by a study showing that depletion of macrophages locally in *mdx* leg muscles from 10 to 12 weeks of age exacerbated dystrophic changes with decreased myofiber formation and increased fat deposition and fibrosis (Madaro et al., 2019). Macrophage depletion impaired proliferation and differentiation of myogenic progenitors and caused adipogenic conversion of satellite cells (Madaro et al., 2019). The Ly6C^hi^ macrophages in the *mdx* leg muscles at 8–10 weeks of age, however, contribute to fibrosis (Juban et al., 2018). They produce latent TGF-β1 due to a high level of latent-TGF-β-binding protein 4 (LTBP4) synthesis, and the latent TGF-β1 is subsequently activated by FAP-derived TGF-β-activating enzymes to promote fibrosis (Juban et al., 2018). Activation of AMPK, which promotes the pro-inflammatory to anti-inflammatory phenotype switch of macrophages (Mounier et al., 2013; McArthur et al., 2020), downregulated LTBP4 expression and TGF-β1 production, leading to decreased fibrosis and improved muscle function (Juban et al., 2018).

Macrophages in the *mdx* diaphragm might be different from those in the *mdx* limb muscles, as the diaphragm undergoes persistent inflammation and progressive fibrosis, while the limb muscles do not (Stedman et al., 1991; Dupont-Versteegden and McCarter, 1992; Zhou et al., 2006; Huang et al., 2011). One study showed that intramuscular fibrocytes, a subset of collagen-producing Ly6C^lo^ macrophages, were more pro-inflammatory and pro-fibrotic in the *mdx* ^*5cv*^ diaphragm than in the *mdx* ^*5cv*^ quadriceps at 14 weeks of age (Wang et al., 2016). But the comprehensive comparison of macrophages between diaphragm and limb muscles is still lacking.

### 4.3 Blocking Monocyte/Macrophage Recruitment by Targeting CCR2 Signaling Provides Transient Benefits in Mdx Mice, Potentiating a Role of Skeletal Muscle-Resident Macrophages

Since macrophages contribute to muscular dystrophy pathology, blocking their recruitment becomes a potential strategy to ameliorate the disease. Blocking macrophage infiltration by genetic ablation or pharmacological inhibition of CCR2 indeed improved muscle pathology and function in the *mdx* diaphragm at early stages (6 and 12 weeks) (Mojumdar et al., 2014). However, the beneficial effects are transient and lost at late stages. CCR2 deficiency in *mdx* ^*5cv*^ mice reduced diaphragm muscle damage and fibrosis and improved diaphragm muscle regeneration and function at 14 weeks but not 6 months (Zhao et al., 2017). Analysis of macrophage recruitment revealed that CCR2 deficiency diminished intramuscular Ly6C^hi^ macrophages at all stages, but reduced Ly6C^lo^ macrophages only at the early stages (4 and 9 weeks) but not the late stages (14 weeks or 6 months) (Zhao et al., 2017). The recovery of Ly6C^lo^ macrophages and the concurrent progression of diaphragm muscle dystrophy at the later stages suggest that the Ly6C^lo^ macrophages are also pathogenic. Therefore, targeting Ly6C^hi^ macrophages alone is not sufficient, and the Ly6C^lo^ macrophages must also be targeted simultaneously. To achieve this, one question must be answered first: where do these Ly6C^lo^ macrophages originate from in the absence of CCR2?

In the absence of CCR2, intramuscular Ly6C^lo^ macrophages may originate from Ly6C^lo^ monocyte recruitment and/or resident macrophage expansion. Since the chemotaxis of Ly6C^lo^ blood monocytes requires CX3CR1 (Charo and Ransohoff, 2006), and the development of Ly6C^lo^ blood monocyte requires Nur77 (Hanna et al., 2011), additional targeting of these two molecules in the *mdx*/*Ccr2* ^*−/−*^mice would help answer whether Ly6C^lo^ blood monocytes are recruited in the *mdx*/*Ccr2* ^*−/−*^ mice, and whether this recruitment contributes to the recovery of Ly6C^lo^ macrophages. Lineage tracing would help determine whether resident macrophage expansion occurs, and whether resident macrophages also regulate muscular dystrophy.

## 5 Resident Macrophages May Play Active Roles in Skeletal Muscle Injury Repair

Following the identification of skeletal muscle-resident macrophages in the steady state, one question arises as to what roles these cells play during injury repair. One early study in rats suggests that muscle resident macrophages do not phagocytose degenerating muscle fibers; they instead act as sentinels activated by damage-associated molecular patterns (DAMPs) during injury to facilitate the recruitment of circulating leukocytes (McLennan, 1993). However, this is contradicted by a recent study showing that resident macrophages in skeletal muscle sense and cloak tissue microlesions to prevent excessive tissue damage under physiological and disease conditions (Uderhardt et al., 2019). Early depletion of resident macrophages in *mdx* mice leads to premature onset of muscle disease featured by increased neutrophil infiltrates (Uderhardt et al., 2019), suggesting that resident macrophages may protect muscle from inflammatory damage at the early stage in this chronic disease model. The contradictory roles might be partially attributed to the existence of different subsets of muscle resident macrophages. Studies of resident macrophages in heart seem to support this hypothesis: following myocardial injury, the CCR2^+^ subset promotes recruitment of neutrophils (Li et al., 2016) and monocytes (Bajpai et al., 2019), while the CCR2^-^ subset inhibits monocytes recruitment (Bajpai et al., 2019). A subset-specific analysis is yet to be done in acutely and chronically injured skeletal muscle.

Following acute skeletal muscle injury, macrophage infiltration is required for the injury repair, suggesting that resident macrophages fail to compensate for the pro-regenerative functions of the inflammatory macrophages (Lu et al., 2011b). During chronic injury in the *mdx* ^*5cv*^/*Ccr2* ^*−/−*^ mice, however, macrophage-mediated inflammation is only compromised at the early stages but recovers at the later stages (Zhao et al., 2017), suggesting that resident macrophage expansion may occur with time to compensate for the lack of recruited inflammatory macrophages. Whether this is true requires further studies. Despite of the scattered evidence, the roles of resident macrophages and their functional subsets in both acute and chronic skeletal muscle injuries are largely unexplored. Studies to specifically target resident macrophages are to be conducted to understand the functions of these cells in muscle inflammation, fibrosis, and regeneration.

## 6 Conclusion

Recent years have seen increasing evidence that macrophages actively regulate diverse physiological and pathological processes. This extraordinary ability relies on their high plasticity in response to tissue environmental changes. Following acute skeletal muscle injury, infiltrating macrophages respond to the changes in intramuscular microenvironment, switching their functional phenotypes in a spatiotemporal manner to promote injury repair. The sequential presence of differentially activated macrophages has been proven vital for well-coordinated, satellite cell-mediated muscle regeneration. In a chronic disease such as DMD, the spatiotemporal activation of macrophages is disrupted, and some macrophages become detrimental, resulting in aberrant muscle regeneration and contributing to the disease progression. Macrophage manipulation, either by blocking their accumulation or by modulating their function, will be likely beneficial to the treatment. To this end, further understanding of the origins, functions, and activation mechanisms of macrophages is required. Advanced technologies, such as lineage tracing and single-cell transcriptome analysis, will continue to help generate valuable insights. It is worth noting that most of the knowledge reviewed here is from animal studies. The knowledge of macrophage contribution to the homeostasis and injury repair in human skeletal muscle is still lacking. Nevertheless, the knowledge gained from animal studies is instructive, which may facilitate future studies of human skeletal muscle. This line of research may eventually develop macrophage-based therapies to promote skeletal muscle injury repair.
